# COVID-19 Vaccination among a Population Experiencing Homelessness: A Survey in Italy

**DOI:** 10.3390/vaccines10122118

**Published:** 2022-12-11

**Authors:** Giorgia Della Polla, Grazia Miraglia del Giudice, Annalisa Napoli, Lucio Folcarelli, Italo Francesco Angelillo

**Affiliations:** 1 Department of Public Health and Laboratory Services, Teaching Hospital of the University of Campania “Luigi Vanvitelli”, Via Luciano Armanni 5, 80138 Naples, Italy; 2 Department of Experimental Medicine, University of Campania “Luigi Vanvitelli”, Via Luciano Armanni 5, 80138 Naples, Italy

**Keywords:** COVID-19, homeless, Italy, knowledge, vaccination

## Abstract

The purposes of this cross-sectional study were to determine the knowledge, attitudes, and behaviors about COVID-19 and its vaccination among 313 individuals experiencing homelessness in Italy and to identify the associated factors. A total of 20.5% identified the virus as a causative agent for COVID-19 and 44.2% identified how the SARS-CoV-2 infection wastransmitted. Those living in homeless shelters were more likely to have this knowledge. Concerns about the safety of the COVID-19 vaccine werehigher in those who were younger, with secondary school as the highest level of education, who practiced Christianity, and who did not believe that COVID-19 was a severe disease. A total of 83.9% received the vaccination. Those who were older, who had correct knowledge, whoperceived to be at a higher risk of getting the disease, and who had a lower concern about the vaccine side effects were more likely to have received the vaccination. The primary reasons for accepting the COVID-19 vaccine were that it wasa preventive measure and that it wasmandatory; those unvaccinated indicated, as the main reasons, a fear of side effects and that it wasnot useful. A relationship and communication between healthcare professionals and this hard-to-reach population are needed, with the implementation of educational and information programs.

## 1. Introduction

As of 15 November 2022, more than 633 million cases have been diagnosed with coronavirus disease 2019 (COVID-19), with more than 6.6 million deaths around the world [1]. Italy has seen one of the highest infection incidences and related deaths among the European countries [2,3]. The public health control measures adopted in Italy, as in many other countries, such as wearing a mask, physical distancing, hand hygiene, SARS-CoV-2 testing, and lockdowns initially contributed to prevent the spread of the disease and, therefore, reduced the number of cases. Moreover, it is important to acknowledge that the epidemiologic evidence has suggested that the available vaccines are the most effective public health interventions for mitigating the impact of the COVID-19 pandemic and for preventing symptomatic SARS-CoV-2 infections and reducing COVID-19-related hospitalizations, complications, and mortality [4,5,6,7]. ACOVID-19 vaccination program was launched in Italy, as in many other countries, in December 2020 and it was prioritized for healthcare workers, residents of nursing homes, the elderly, and essential workers. In March 2021, the vaccine was available to all adults [8].

Prior research has well-documented the disparities in healthcare access among minority communities—for example, people experiencing homelessness—with vaccination coverage rates that are very low [9,10].Therefore, this population may be at an elevated risk of COVID-19 and the associated illness, hospitalizations, and deaths, but little is available about the COVID-19 vaccination uptake and the correlates among them [11,12]. Previous investigations have largely focused onthe attitudes and behaviors regardingCOVID-19 vaccinations among different groups [13,14,15,16,17,18]. However, this topic among the homeless is poorly explored [19,20,21,22,23,24]. Thus, asit is critical to acquire this information and to fill a gap in the existing research, the purposes of this current study were to determine the COVID-19 vaccine uptake among a homeless population in Italy and to identify the potential associated factors.

## 2. Materials and Methods

This survey formed part of a large ongoing COVID-19 vaccination research activity undertaken among different groups of people living in Southern Italy [14,16,17,18,25,26,27].

### 2.1. Study Design, Setting, and Participants

This cross-sectional survey was performed between June and October 2022. The study participants were all individuals experiencing homelessness who attendedsix randomly selected centers known to be relevant in providing accommodation and catering as well as health, social, and practical support in the geographic area of Naples and Salerno in the Southern part of Italy. Homeless peoplewere defined as aged 18 years old or over, who attended these centers, and who were able to comprehend all questions in Italian without assistance. No specific exclusion criteria were applied.

### 2.2. Procedure

An invitation letter introducing the research topic and information about the study were sent to the managers of the centers to ask for their collaboration. After obtaining permission to conduct the study, four trained investigators visited these centers several times at different time periods, including on weekends and evenings, and all homeless attendees at the time of the study were approached. The attendees were asked about their interest in participating in the study and were provided with information regarding its background and objectives; that participation was voluntary, anonymous, and confidential; and that all questions were compulsory. It was also highlighted that they were able to withdraw from participation at any point without any explanation required. Those who expressed an interest were requiredto sign an informed consent form for their participation prior to the survey administration. Face-to-face interviews were conducted by the four investigators at the centers. No financial or other incentives were provided for participation in the survey.

The Ethics Committee of the Teaching Hospital of the University of Campania “Luigi Vanvitelli” reviewed and approved the protocol and the questionnaire.

### 2.3. Survey Instrument

The interview questionnaire was basedon the contents of previous survey instruments forthis field used by a fewmembers of the research group among different populations [13,15,16,17,24,25,26]. The questionnaire was pilot-tested among ten non-selected individuals prior to the initiation of the study for general acceptability in terms of accuracy, clarity, and consistency. Data from the pilot study were not included in the final analysis.

The questionnaire was divided into five sections. The first section was related to the sociodemographic and general characteristics, including gender, age, nationality, marital status, education, living condition, having been infected with SARS-CoV-2, and knowing someone who had been infected with SARS-CoV-2. The second section contained three questions to assess knowledge about the SARS-CoV-2 infection and COVID-19 vaccine. The third section consisted of eight statements regarding attitudes and beliefs about the SARS-CoV-2 infection and COVID-19 vaccine. Responses were collected on a ten-point Likert scale, where “one” was “not at all” and “ten” was “very much”. The fourth section consisted of sixquestions related to thereceipt of a COVID-19 vaccination and the reason(s) for their decision. Those unvaccinated were asked whether they intended to vaccinate against COVID-19 and the reason(s) in favor or against the vaccination. The last section included two questions regarding the sources from which they had obtained theirinformation relating to the COVID-19 vaccination; they had multiple choices for the responses. Finally, there was a question on whether they would like to receiveadditional information.

### 2.4. Statistical Analysis

First, the descriptive statistics to detail the total sample werepresented as the frequencies, proportions, and means, with standard deviations when appropriate. Second, chi-squared tests and Student’s t-tests were used to examine the association between the categorical or continuous predictors and the categorical and continuous variables, respectively. Third, all independent variables with a *p*-value less than or equal to 0.25 at the bivariate analysis were included in the multivariate linear and logistic regression models. Three multivariate models were designed to address the possible association between the different variables and the following dependent variables: knowledge of the virus as a causative agent for COVID-19 and that a SARS-CoV-2 infection is transmitted during close contact and byairborne (Model 1); concern about the safety of the COVID-19 vaccine (Model 2); and having received the COVID-19 vaccine (Model 3). The following independent variables were selected because they potentially related to all dependent variables: gender (male = 0; female = 1); age, in years (continuous); marital status (unmarried/separated/divorced/widowed = 0; married/cohabitant = 1); Italian nationality (no = 0; yes = 1); practicing Christianity (no = 0; yes = 1); level of education (none = 0; middle school = 1; secondary = 2; baccalaureate/graduate degree= 3); living in a homeless shelter (no = 0; yes = 1); at least one chronic medical condition (no = 0; yes = 1); having been infected by SARS-CoV-2 (no = 0; yes = 1); knowing at least one relative/friend who had been infected by SARS-CoV-2 (no = 0; yes = 1); and requirementof additional information on COVID-19 (no = 0; yes = 1). The following variables were also included in the different models: having received information regarding the COVID-19 vaccination from physicians (no = 0; yes = 1) in Models 1 and 3; knowledge of the virus as a causative agent for COVID-19 and that a SARS-CoV-2 infection is transmitted during close contact and byairborne (no = 0; yes = 1); perceived concern of the severity of COVID-19 (continuous) and perceived concern of being infected by SARS-CoV-2 (continuous) in Models 2 and 3; having received information regarding the COVID-19 vaccination from mass media/internet sites/social networks (no = 0; yes = 1) in Model 2; and concern about the safety of the COVID-19 vaccine (continuous) in Model 3. A stepwise regression was used for the selection of the independent variables in the final model by adding or removing the potential explanatory variables in succession with a threshold of *p* = 0.2 and *p* = 0.4, respectively. The results from the logistic regression models were expressed in terms of odds ratios (ORs) with associated 95% confidence intervals (CIs) whereas the beta regression coefficient (β) was used withthe linear regression models. All reported *p*-values were based on two-tailed tests and a *p*-value equal to or less than 0.05 indicated a statistical significance. The data were managed and analyzed using STATA version 15.1 software.

## 3. Results

### 3.1. Participant Characteristics

Across the 6 centers, and from the total of 330 individuals who were approached, 313 agreed to participate, providing a response rate of 89%. Table 1 details the principal characteristics of the respondents. Most were male (78.5%), the mean age was 49.3 years, one-third (29.8%) were single, half (50.8 %) were Italian, 50.8 % lived in a homeless shelter, only 18.3% reported having had a COVID-19 infection, a COVID-19 infection in a friend/family member was declared by 33.2% respondents, and 34.7% had one or more comorbidities.

### 3.2. Knowledge about COVID-19

The sample demonstrated a low level of knowledge about COVID-19 and its vaccination in most of the questions. Only 20.5% correctly identified the virus as a causative agent for COVID-19, more than one-third (44.2%) correctly knew that a SARS-CoV-2 infection is transmitted during close contact and byairborne, and only 43.1% declared that the vaccination was mandatory. Overall, only 13.7% participants demonstrated knowledge of COVID-19 by correctly answering all three questions. Multivariate linear and logistic regression analyses were conducted to identify among the different characteristics those who were significantly associated with the various outcomes of interest; the results are reported in Table 2. The results of the first multivariate logistic regression model showed that those living in homeless shelters (OR = 1.95, 95% CI = 1.04–3.68) were more likely to correctly identify the virus as a causative agent for COVID-19 and how a SARS-CoV-2 infection is transmitted (Model 1).

### 3.3. Health Beliefs regarding COVID-19

In the context of the health beliefs of the respondents regarding COVID-19, which were measured using a ten-point Likert scale ranging from “one” representing “not at all” to “ten” representing “very much”, the overall mean value of the belief that this wasa severe disease was 7.1, with more than one-third (39.7%) indicating a value of ten. When the participants were asked if they perceived themselves to be susceptible to this disease, the mean score was 5.8; 25.5% and 28.1% responded “one” and “ten”, indicating a very low and very high concern, respectively. Regarding the last statement, a very low concern was expressed about the side effects of the vaccination, with an overall mean value of four. Almost half (48.2%) had no concern at all and only 14.4% responded with a value of ten. The multivariate linear regression model showed that four characteristics were significantly related to the level of concern of the respondents about the safety of the COVID-19 vaccine. The level of concern was significantly higher in those who were younger, in those with secondary school as the highest level of education compared with those with no education, in those who practiced Christianity, and in those who did not believe that COVID-19 was a severe disease (Model 2 in Table 2).

### 3.4. COVID-19 Vaccine Behavior and Willingness

Of the 313 respondents, 83.9% declared a compliance with the current vaccination schedule. In the final multivariate logistic regression model performed with the outcome variable of the COVID-19 vaccine uptake, four independent characteristics resulted in a statistically significant association. Respondents who were older (OR = 1.04, 95% CI = 1.01–1.07), those who correctly identified the virus as a causative agent for COVID-19 and how a SARS-CoV-2 infection is transmitted (OR = 3.04, 95% CI = 1.04–8.94), those who perceived themselves to be at a higher risk of getting the disease (OR = 1.21, 95% CI = 1.09–1.35), and those who expressed a lower concern about the side effects of the vaccination (OR = 0.78, 95% CI = 0.71–0.86) were more likely to have received the vaccine against COVID-19 (Model 3 in Table 2). The primary reasons for accepting the COVID-19 vaccine were that it was a preventive measure (55.2%) and that it was mandatory (28.3%) whereas those unvaccinated indicated as their main reasons a fear of side effects (54%) and that it was not useful (10%). Among those who were unvaccinated, less than one-third (29.8%) of the surveyed homeless people reported that they were likely to get vaccinated. Among those who intended to get a COVID-19 vaccine, the main reasons given were the vaccine efficacy (57.1%) and that the disease is severe (42.8%) whereas among those who were undecided (12.8%) or unwilling (57.4%) to get this vaccine, the leading reasons were concerns about the side effects (54.5%) and efficacy (36.4%).

### 3.5. Sources of Information Received

Almost all participants had received information about vaccination against COVID-19 (91.2%). The primary sources through which the respondents had heard about the COVID-19 vaccination were the TV, radio, or newspapers (48.5%), followed by the internet (40.2%). Only one-fourth had heard from physicians (25.3%). Only 11.2% of the respondents wanted additional information about a vaccination against COVID-19.

## 4. Discussion

Despite the large number of studies examining the knowledge, attitudes, and behaviors among different groups of individuals, as far as we know, this is the first survey that has sought to understand the knowledge, attitudes, and behaviors of homeless people about COVID-19 and its vaccination conducted in Italy. This study adds information by examining a wider range of correlates than those previously reported in the literature to date.

The sample in the present research revealed a lack of basic knowledge. Although the majority were aware of the vaccination, less than one-fifth knew about the cause of COVID-19 and, most importantly, less than half correctly answered the question about the possible transmission modes. As knowledge about COVID-19, as well as attitudes regarding its vaccination, are well-known critical instruments for preventing the spread of the infection, this lack of knowledge is of concern because it may limit the application of the public health measures recommended to mitigate the transmission of SARS-CoV-2 to healthy people in different settings. Therefore, in this context, the low level of knowledge warrants attention and underscores the need for educational campaign efforts to disseminate adequate and accurate information to raise COVID-19-related knowledge among this group. The low level demonstrated by the participants in this survey was not just a phenomenon of this sample as it has already been documented for hard-to-reach populations regarding a variety of vaccinations [28,29]. Furthermore, in this study, the analysis of the determinants of knowledge showed that the status of knowing that the virus is a causative agent for COVID-19 and that a SARS-CoV-2 infection is transmitted during close contact and byairborne was significantly higher only among those who lived in a homeless shelter.

Regarding the attitudes towards the COVID-19 disease and its vaccination, the participants perceived themselves to be not particularly susceptible to this disease, with less than one-third indicating a very high concern. A very low concern was expressed about the side effects of the vaccination and almost half of the respondents had no concern at all. The results of the multivariate analysis established that Christian respondents were more likely to express a higher concern compared with those who were not Christian. This significant impact of religion on the concern about the safety of the COVID-19 vaccine has been already reported in the literature, showing Christianity to be one of the strongest predictors of hesitancy and also negatively associated with having received or planning to get a COVID-19 vaccine [30,31]. This analysis further established that the age and the level of education of the respondents were other key determinants of the perception towards the vaccination, as homeless people of a younger age and with no education were more likely to have a higher level of concern than those who were older and who had secondary education as the highest level. Possible explanations may be due to the high frequency of cases of COVID-19 and the complications among those who are older; those with a lower level of educational attainment may find it more difficult to acquire information as well as understanding the vaccination. It is encouraging that, despite safety concerns, the vast majority of the sample had been immunized.

Despite the poor level of knowledge among this study population, the vast majority (82.8%) self-reported that they had received the COVID-19 vaccine and among those unvaccinated, less than one-third (30.8%) indicated that they were willing to get vaccinated. The observed COVID-19 vaccine uptake was slightly lower than the value of 89.4% observed in the Italian general population [32], but it was considerably higher than those found in studies among homeless people also conducted in other countries. In Italy, in homeless settlements, the uptake ranged from 13.3% to 35.9% [22]; in the United States, the fully vaccinated coverage was 0.6% among homeless shelter residents [23], 34.6% in people experiencing homelessness from 18 years of age [19] and 18.6–44.5% from 16 years of age [24]; and in Canada, 47.7% of individuals with a recent history of homelessness had received two doses [21] and 63.6% of people experiencing homelessness had received two or more doses [20]. It is important to underline that the multivariate logistic regression analysis identified several key variables influencing the COVID-19 vaccine uptake in the sample. These included mixed aspects of the sociodemographic characteristics, knowledge, and attitudes towards the disease and its vaccination. First, homeless people who were of an older age were more likely to be vaccinated; potential explanations include the established observation that older adults are at a particular risk of having a severe infection and of dying as a result of the disease. Second, respondents who correctly identified the causative agent for COVID-19 and the ways of transmission were three times more likely to have received the vaccine than those who did not have this knowledge. This finding confirmed the need to impart knowledge and was consistent with the existing evidence, which showed that an adequate knowledge was associated with a higher vaccine uptake [33,34,35,36]. Third, homeless people with a higher level of perceived susceptivity to this disease and those with a lower level of concern about the side effects of this vaccination were associated with higher odds of a vaccine uptake. Interventions are needed to reinforce the clear beneficial effects of the vaccination and to prevent misconceptions about its safety and efficacy.

Participants most commonly mentioned the belief that the vaccine was an important preventive measure as the main reason for having received the vaccine whereas, unsurprisingly, concerns about the potential side effects and that it was not useful were identified as the leading reasons for a COVID-19 vaccine refusal. These results, which were in concordance with previous similar surveys conducted worldwide among different groups of individuals [14,25,33,37,38], underscore the need for intensified educational activities on the COVID-19 vaccine, which can address these incorrect beliefs. Moreover, it is important to note that a substantial minority (7.7%) reported that they had received the vaccine from the recommendation of a healthcare provider. This very low value underlined that public health interventions at the provider and healthcare system level are needed to educate providers on the importance of recommending the vaccine to this hard-to-reach group of people. Furthermore, previous research has clearly shown a significant positive influence of receiving a vaccination recommendation by healthcare professionals in individual decision-making regarding the uptake [39,40,41,42,43].

A concerning result of the present survey was that only one-fourth of the participants reported having obtained information about the COVID-19 vaccination from a physician. This finding is crucial because information from physicians, as from other healthcare professionals as well as the recommendations previously mentioned, have been shown in previous studies to improve the level of knowledge, the willingness to accept the vaccination, and the vaccine uptake rates [14,27,44,45,46]. The reason for the lack of using this source might be that homeless people may have more difficulty in accessing a primary care physician or preventive healthcare services and in obtaining health information; therefore, there is a lack of opportunity to improve their level of knowledge. This is consistent with the broader literature showing that the physician–patient relationship acts as a barrier for underserved communities, and such individuals may access healthcare services less often [47,48,49] as well as the observation that the COVID-19 pandemic has negatively affected access to health-related information and sources of healthcare worldwide [50,51,52,53]. On the other hand, the surveyed respondents indicated that the most common information resources used were mass media and the internet. This was another concerning finding because many international studies have determined that inadequate information about the vaccine is commonly spread through these sources, which leads to misconceptions about the safety and efficacy of the COVID-19 vaccine, a lower level of knowledge, and an unwillingness to accept vaccines for themselves and for their children [54].

There are a few potential methodological limitations that should be borne in mind when interpreting the results of the present survey. First, the survey adopted a cross-sectional design that precluded the ability to infer cause-and-effect conclusions of the identified associations between the predictors and the outcomes. Second, the data were collected only within one geographic site; thus the generalizability of the findings to the entire homeless population in Italy should be cautiously interpreted. Third, the data were based on the self-reported information of the participants, which may have beenprone to recall bias and, therefore, less accurate. Fourth, data might have been misreported, either intentionally or not, to share socially desirable or undesirable perceptions and practices. This might have been especially true when asked if they had received the vaccine; this information was not integrated with the COVID-19 immunization registry to verify whether they had received it. However, all responses were confidential and anonymous, and this may have had positively impacted on the honesty of the responses. Despite the mentioned potential limitations, the current results provided insights and added important values to the field.

To sum up, the results of the survey showed that the homeless people surveyed did not have an adequate level of knowledge about COVID-19 and its vaccination; the majority had received the vaccination, but only a very small number from the recommendation of a healthcare provider; and concerns about the potential side effects and usefulness of the vaccine were the main reasons preventing vaccinations. Based on these findings, it is mandatory to improve the relationship and the communication between healthcare professionals and this hard-to-reach population; to implement educational and information programs to improve the level of knowledge; and to address the misconceptions about COVID-19 asthis vaccination is imperative for reducing the frequency of the disease and the clinical burden.

## Figures and Tables

**Table 1:** Main sociodemographic and general characteristics of the sample.

Characteristics	N	%
Age, years	49.3 ± 17.2 (18–86) *	
Gender		
Male	244	78.5
Female	67	21.5
Marital status		
Married/cohabitant	214	70.2
Unmarried/separated/divorced/widowed	91	29.8
WHO region of origin		
European (Italy)	159	50.8
Asian	69	22.2
African	51	16.3
European (other than Italy)	28	8.9
Others	6	2.8
Religion		
Christian	182	58.1
Muslim	87	28.9
Others	44	13
Level of education		
None	79	26.8
Middle school	81	27.5
Secondary	79	23.7
Baccalaureate/graduate degree	65	22
Living conditions		
Homeless shelter	158	50.8
Relatives’ or friends’ place	75	24.1
Streets	65	20.2
Other	13	4.9
Having at least one chronic medical condition		
No	203	65.3
Yes	108	34.7
Having been infected by SARS-CoV-2		
No	254	81.7
Yes	57	18.3
At least one relative/friend infected by SARS-CoV-2		
No	207	66.8
Yes	103	33.2
Having been vaccinated against COVID-19		
No	50	16.1
Yes	261	83.9

Number for each item may not add up to total number of the study population due to missing values. * Mean ± standard deviation (range).

**Table 2:** Results of the multivariate logistic regression analysis showing the determinants of the different outcomes of interest.

Variable	OR	SE	95% CI	*p*-Value
**Model 1.** Knowledge of the virus as a causative agent for COVID-19 and that a SARS-CoV-2 infection is transmitted during close contact and byairborne. Log likelihood = −133.88, χ^2^ = 22.02 (7 df), *p* < 0.0001				
Accommodation in homeless shelter	1.95	0.63	1.04–3.68	0.037
Having at least one chronic medical condition	1.73	0.54	0.93–3.21	0.082
Male	0.44	0.21	0.17–1.11	0.084
Having been infected by SARS-CoV-2	1.67	0.61	0.81–3.43	0.163
Level of education				
None	1.00 *			
Middle school	1.58	0.66	0.71–3.57	0.264
Secondary	1.85	0.84	0.76–4.49	0.172
Baccalaureate/graduate degree	0.59	0.33	0.2–1.78	0.353
	**β Coeff.**	**SE**	***t***	***p*-value**
**Model 2.** Higher perceived concern about the safety of the COVID-19 vaccine. F(12, 262) = 5.17, *p* < 0.0001, *R^2^* = 19.1%, adjusted *R^2^* = 15.4%				
Christian	1.74	0.53	3.3	0.001
Lower perception of the severity of COVID-19	−0.24	0.81	−3.05	0.003
Younger	−0.03	0.14	−2.24	0.026
Level of education				0.045
None	1.00 *			
Middle school	−0.78	0.51	−1.54	0.124
Secondary	−1.19	0.59	−2.02	0.045
Baccalaureate/graduate degree	−0.72	0.62	−1.17	0.245
Knowing at least one relative/friend who had been infected by SARS-CoV-2	0.85	0.47	1.82	0.07
Having at least one chronic medical condition	0.66	0.43	1.53	0.126
Female	0.73	0.51	1.42	0.158
Having been infected by SARS-CoV-2	0.78	0.53	1.35	0.18
Higher perceived concern of being infected by SARS-CoV-2	0.08	0.07	1.19	0.236
Italian	0.61	0.54	1.12	0.264
	**OR**	**SE**	**95%CI**	***p*-value**
**Model 3.** Having received the COVID-19 vaccine. Log likelihood = −106.13, χ^2^ = 55.17 (6 df), *p* < 0.0001				
Lower perceived concern about the safety of the COVID-19 vaccine	0.78	0.41	0.71–0.86	<0.001
Higher perceived concern of being infected by SARS-CoV-2	1.21	0.07	1.09–1.35	<0.001
Older	1.04	0.01	1.01–1.07	0.002
Correct knowledge about the causative agent for COVID-19 and how a SARS-CoV-2 infection is transmitted	3.04	1.67	1.04–8.94	0.043
Christian	2.06	0.91	0.87–4.91	0.102
Male	0.51	0.22	0.22–1.21	0.129

* Reference category.

## Data Availability

The anonymous data presented in this study are available on request from the corresponding author.
